# Effect of Vitamin B Deprivation during Pregnancy and Lactation on Homocysteine Metabolism and Related Metabolites in Brain and Plasma of Mice Offspring

**DOI:** 10.1371/journal.pone.0092683

**Published:** 2014-04-02

**Authors:** Vanessa Cavalcante da Silva, Leandro Fernandes, Eduardo Jun Haseyama, Ana Luiza Dias Abdo Agamme, Elvira Maria Guerra Shinohara, Maria Tereza Cartaxo Muniz, Vânia D'Almeida

**Affiliations:** 1 Department of Pediatrics, Universidade Federal de São Paulo, São Paulo, São Paulo, Brasil; 2 Department of Psychobiology, Universidade Federal de São Paulo, São Paulo, São Paulo, Brasil; 3 School of Pharmaceutical Sciences of University of Sao Paulo, São Paulo, São Paulo, Brasil; 4 Pediatrics Hematology and Oncology Center, Biological Science Institute, Universidade de Pernambuco, Recife, Pernambuco, Brasil; Idaho State University, United States of America

## Abstract

Epidemiological and experimental studies indicate that the altered fetal and neonatal environment influences physiological functions and may increase the risk of developing chronic diseases in adulthood. Because homocysteine (Hcy) metabolic imbalance is considered a risk factor for neurodegenerative diseases, we investigated whether maternal Vitamin B deficiency during early development alters the offspring's methionine-homocysteine metabolism in their brain. To this end, the dams were submitted to experimental diet one month before and during pregnancy or pregnancy/lactation. After birth, the offspring were organized into the following groups: control (CT), deficient diet during pregnancy and lactation (DPL) and deficient diet during pregnancy (DP). The mice were euthanized at various stages of development. Hcy, cysteine, glutathione (GSH), S-adenosylmethionine (SAM), S-adenosylhomocysteine (SAH), folate and cobalamin concentrations were measured in the plasma and/or brain. At postnatal day (PND) 0, total brain of female and male offspring exhibited decreased SAM/SAH ratios. Moreover, at PND 28, we observed decreased GSH/GSSG ratios in both females and males in the DPL group. Exposure to a Vitamin B-deficient diet during the ontogenic plasticity period had a negative impact on plasma folate and brain cortex SAM concentrations in aged DPL males. We also observed decreased plasma GSH concentrations in both DP and DPL males (PND 210). Additionally, this manipulation seemed to affect the female and male offspring differently. The decreased plasma GSH concentration may reflect redox changes in tissues and the decreased brain cortex SAM may be involved in changes of gene expression, which could contribute to neurodegenerative diseases over the long term.

## Introduction

Homocysteine (Hcy) is a sulfur-containing amino acid derived from the metabolism of methionine [1], [2]. First, the methionine adenosyltransferase enzyme produces an intermediate metabolite called S-adenosylmethionine (SAM), which has an important biological function as methyl donor in a multitude of cellular methylation reactions [3]. After the transmethylation reaction by the action of specific methyltransferases, SAM is converted into S-adenosylhomocysteine (SAH). Interestingly, SAH is an inhibitor of SAM-dependent methyltransferases activity, and the ratio SAM/SAH is used as an index of cellular methylation potential. SAH is then hydrolyzed to adenosine and Hcy by the enzyme SAH hydrolase [4]. In fact, the reaction catalyzed by SAH hydrolase is reversible, and thermodynamics favors AdoHcy (also named SAH) synthesis rather that Hcy production. So, when Hcy accumulates, SAH will accumulate as well. However, under normal conditions Hcy will be quickly metabolized and the reaction proceeds in the direct way. The Hcy may be then remethylated to methionine by the ubiquitously distributed methionine synthase (MS), or by betaine-homocysteine methyltransferase (BHMT) in the liver and kidney of some species. MS utilizes 5-methyltetrahydrofolate and cobalamin as its methyl donor and cofactor, respectively, whereas BHMT employs betaine, which is produced during choline oxidation as well as being provided by the diet [5]. Additionally, Hcy can be catabolized through its transsulfuration, through a series of reactions that end with the production of cysteine, which may be further used in glutathione (GSH) production. Accordingly, increasing evidence supports the importance of the transsulfuration pathway of Hcy in the maintenance of the redox homeostasis [6].

Hcy levels can vary considerably among individuals depending on genetic, dietary and environmental factors, and elevated plasma concentrations have been identified as a risk factor for neurodegenerative diseases [7], [8]. The disturbance of maternal and fetal Hcy metabolism attributable to folate or cobalamin shortage has been shown to play a role in the etiology of recurrent early pregnancy loss, placental abruption and preeclampsia [9]–[11]. In progeny, hyperhomocysteinemia (hHCY) effects have been associated with premature birth, intrauterine growth retardation, neural tube defects and fetal death [12], [13].

Evidence from observational and experimental studies suggests a link between adverse exposures in early life, particularly relating to nutrition status (restriction or supplement), and chronic disease susceptibility in adulthood. For example, studies in rats have found that maternal gestational diabetes exposure in the fetal or neonatal period can result in permanent changes in body fat mass and in the hypothalamic neuronal circuitry regulating appetite in the adult brain [14], [15]. According to the developmental origin of health and disease hypothesis, increased susceptibility to disease is partly shaped during fetal programming by the interaction of nutrition and epigenetic mechanisms [16].

Cobalamin and folate deficiency in pregnancy is relatively common, especially in some countries of sub-Saharan Africa, northern Europe and Brazil [17]–[20]. A study on rats with hHcy during pregnancy indicated that maternal folate status alters the homeostasis of one-carbon metabolism and the methyl pool, which would in part affect placental DNA methylation [21]. Interestingly, gestational Vitamin B deficiency leads to an accumulation of Hcy with concomitant apoptosis in select brain areas and alters neurobehavioral capacities during rat development [22]. Furthermore, maternal chronic hHcy can impair brain development and, consequently, the cognitive functions of the fetus by increasing neuronal vulnerability to excitotoxicity and oxidative stress [23], [24]. In humans, low maternal folate status during early pregnancy was associated with a higher risk of emotional problems in the offspring, such as anxiety and depression, behavioral withdrawn symptoms and somatic complaints [25]. Despite these studies, little is known about the effects of vitamin B deficiency in fetuses and neonates on their methionine-homocysteine metabolism programming and subsequent consequences to the brain. Considering that exposure to suboptimal nutrition during early development leads to a long-term, age-dependent metabolic response [26], we investigated the effects of a vitamin B-deficient diet during pregnancy or pregnancy/lactation in mice offspring. To this end, we quantified Hcy, cysteine (Cys), GSH, SAM, SAH concentrations and folate and cobalamin cofactors in plasma and/or brain on postnatal day (PND) 0, 5, 28, 90 and 210.

## Results

### Dams

**Figure 1:**
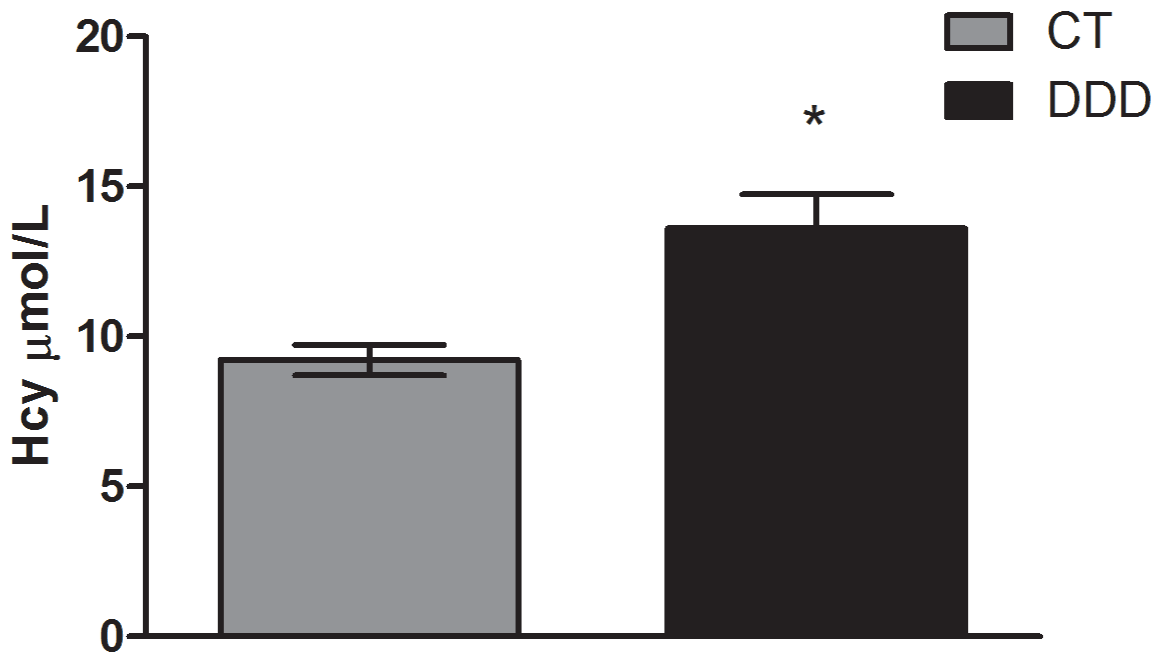
Plasma Hcy concentration in dams after twenty days on an experimental diet. n = 6–8; PND  =  Postnatal day; CT  =  control; DDD  =  dams in deficient diet. *t test*. Data are presented as the mean ± standard error. ^*^ *P*≤0.05.

The dams that received the deficient diet (DDD) had, after 20 days, approximately a 50% increase in plasma Hcy concentrations (13.606 μmol/L) compared to control group (CT) (9.219 μmol/L), *P*<0.001 (Figure 1).

### Offspring

After birth, the offspring were separated into three groups: control (CT), deficient diet during pregnancy and lactation (DPL) and deficient diet during pregnancy (DP). The dams of offspring CT and DPL received, respectively, standard and deficient diet during pregnancy and lactation, and the DP offspring was breastfed by control dams that received the standard diet during lactation.

**Figure 2:**
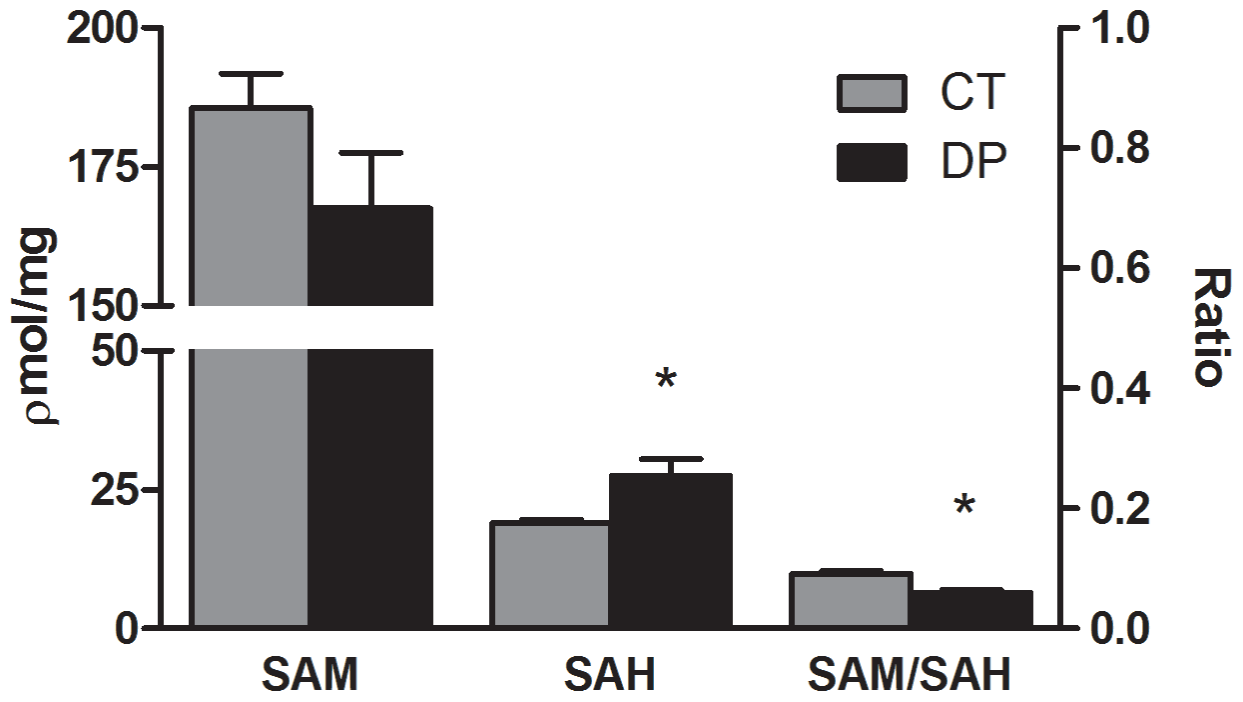
Effects of a Vitamin B-deficient diet during pregnancy on SAM and SAH concentrations and SAM/SAM ratios in the brain of female offspring at PND 0. n = 6–8; PND  =  Postnatal day; CT  =  control; DP  =  deficient diet during pregnancy; SAM  =  S-adenosylmethionine; SAH  =  S-adenosylhomocysteine. *t test*. Data are presented as the mean ± standard error. ^*^ *P*≤0.05.

**Figure 3:**
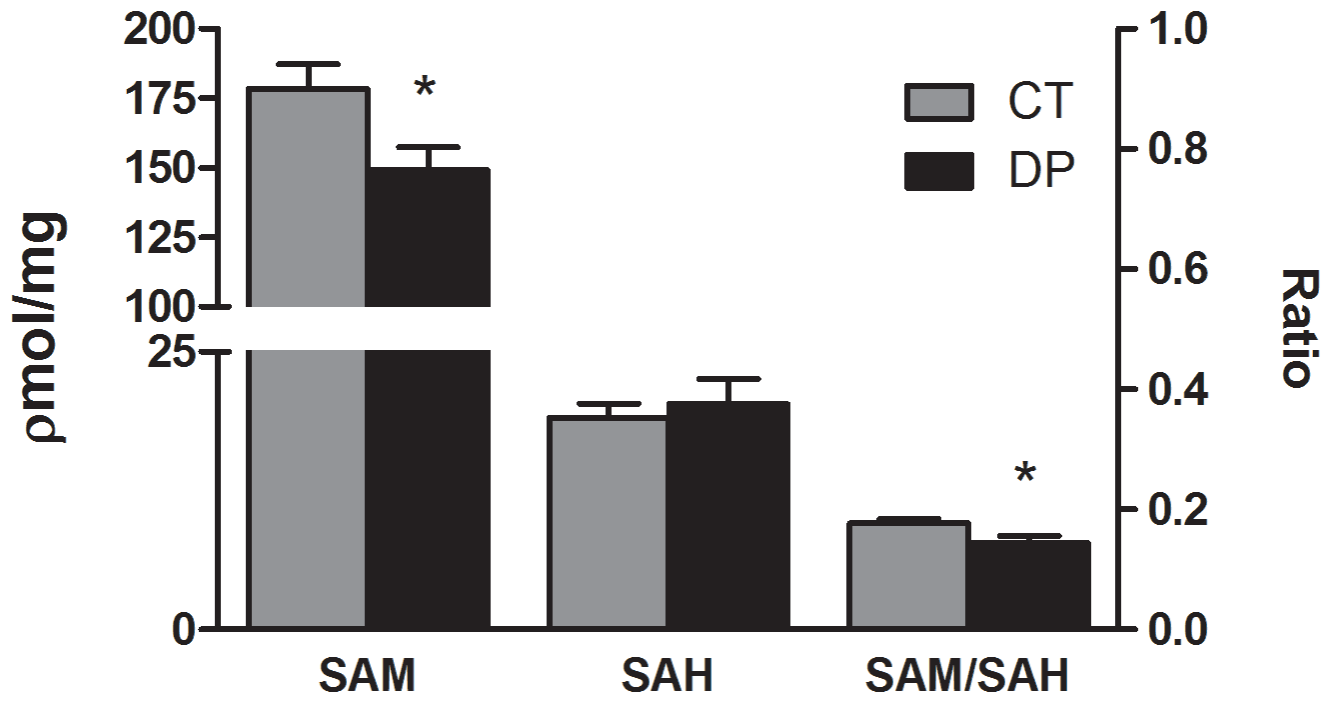
Effects of a Vitamin B-deficient diet during pregnancy on SAM and SAH concentrations and SAM/SAM ratios in the brain of male offspring at PND 0. n = 6–8; PND  =  Postnatal day; CT  =  control; DP  =  deficient diet during pregnancy; SAM  =  S-adenosylmethionine; SAH  =  S-adenosylhomocysteine. *t test*. Data are presented as the mean ± standard error. ^*^ *P*≤0.05.

**Figure 4:**
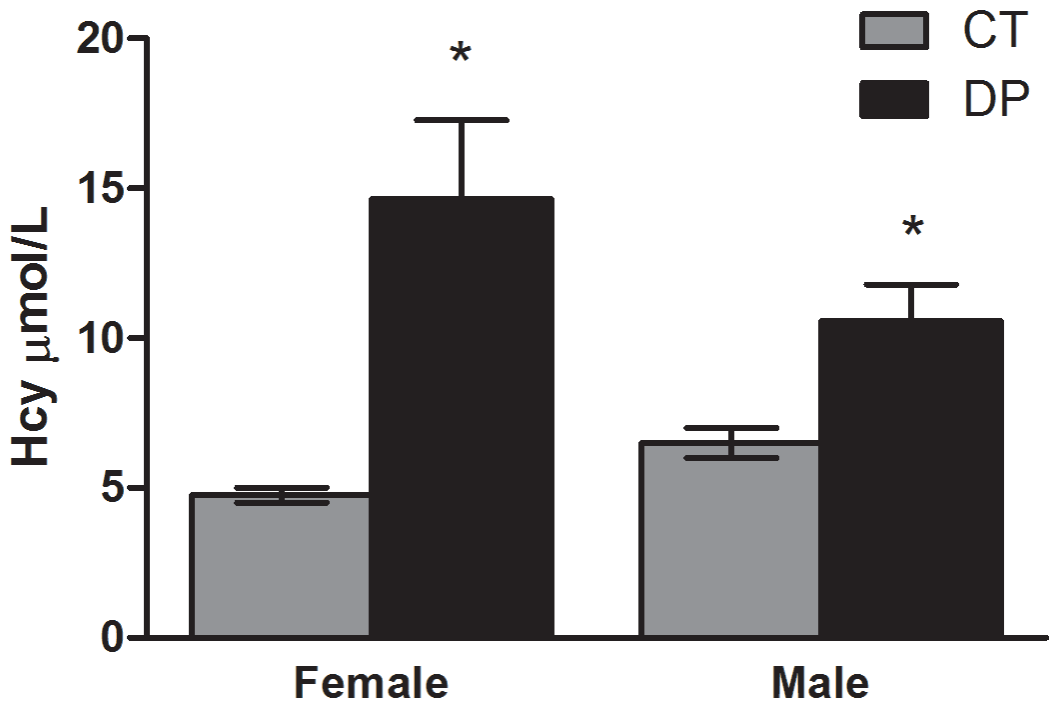
Effects of a Vitamin B-deficient diet during pregnancy and lactation on plasma Hcy concentrations of female and male offspring at PND 5. n = 6–8; PND  =  Postnatal day; CT  =  control; DP  =  deficient diet during pregnancy. *t test*. Data are presented as the mean ± standard error. ^*^ *P*≤0.05.

To evaluate the effect of Vitamin B deficiency during pregnancy on brain methyl metabolism in the offspring, SAM and SAH were measured after birth (PND 0) in the DP and CT groups. The brain tissue of female offspring exposed to the deficient diet exhibited, when compared to CT, increased concentration of SAH (*P* = 0.014) but no significant difference in SAM concentration (*P* = 0.140; Figure 2). Interestingly, the brain analysis of male offspring did not show changes in SAH concentration (*P* = 0.620) but did exhibit a decrease in SAM concentration (*P* = 0.028, Figure 3). However, both females (*P*<0.001) and males (*P* = 0.033) exhibited a decrease in SAM/SAH brain ratios. The concentrations of total GSH (*P* = 0.372 and *P* = 0.841), reduced GSH (*P* = 0.592 and *P* = 0.805), GSSG (*P* = 0.148 and *P* = 0.654) and GSH/GSSG ratios (*P* = 0.185 and *P* = 0.699) were not significantly different in total brain of females and males, respectively (Table 1). In this period, the plasma analyses were not performed because of insufficient material. However, at PND 5 plasma Hcy was quantified and an increase in both female (*P* = 0.001) and male (*P* = 0.013) DPL was observed when compared to the CT group (Figure 4).

**Table 1:** Effects of a Vitamin B-deficient diet during pregnancy on total GSH, reduced GSH and GSSG concentrations, and GSH/GSSG ratios in the brain of offspring at PND 0.

PND 0	Female		Male	
	CT	DP	CT	DP
**Brain total GSH (nmol/mg)**	6.5±0.3	7.0±0.4	6.5±0.3	6.4±0.4
**Brain reduced GSH (nmol/mg)**	4.9±0.1	4.7±0.3	4.9±0.2	5.0±0.3
**Brain GSSG (nmol/mg)**	0.8±0.1	1.2±0.1	0.8±0.2	0.7±0.1
**GSH/GSSG ratio**	7.0±1.0	4.9±1.0	9.5±2.4	8.0±1.3

n = 6–8; PND  =  Postnatal day; CT  =  control; DP  =  deficient diet during pregnancy; GSH  =  glutathione; GSSG  =  oxidized glutathione. *t test*. Data are presented as the mean ± standard error.

Results concerning plasma dosage at PND 28 are summarized in Table 2. When the effects of a deficient diet in PND 28 (weaning) were evaluated, a significant increase in plasma Hcy concentration was observed in the female DPL group when compared to the DP (*P*<0.001) and CT (*P*<0.001) groups. This increase was approximately four times higher than in PND 5, in both female and male offspring. Moreover, the DPL group had a decrease in plasma folate and cobalamin concentrations when compared to the DP (*P*<0.001 and *P* = 0.019) and CT (*P* = 0.001 and *P* = 0.013) groups, respectively. However, there were no changes in plasma Cys (*P* = 0.184) and total GSH (*P* = 0.590) concentrations. Similar results were observed in males, which showed increased Hcy concentration in the DPL group when compared to the DP (*P*<0.001) and CT (*P*<0.001) groups, respectively, and a significant decrease in plasma folate concentration when compared to the DP (*P*<0.001) and CT (*P*<0.001) groups. There was also a decrease in cobalamin concentration when comparing the DPL to CT (*P*<0.008) group. No changes were observed in plasma Cys (*P* = 0.418) and total GSH (*P* = 0.911) concentrations in males.

**Table 2:** Effects of a Vitamin B-deficient diet during pregnancy and pregnancy/lactation on plasma and brain cortex parameters in offspring at PND 28.

PND 28	Female			Male		
	CT	DP	DPL	CT	DP	DPL
**Plasma Hcy (μmol/L)**	9.7±0.6	11.0±1.3	57.8±7.5*^#^	8.7±0.9	10.0±0.9	47.2±5.6*^#^
**Plasma Cys (μmol/L)**	323.2±26.5	347.3±25.7	282.0±22.6	303.9±19.7	302.7±17.0	269.5±24.3
**Plasma GSH (μmol/L)**	109.2±5.4	109.5±6.0	102.0±6.8	113.7±8.7	112.2±6.8	109.2±10.7
**Plasma folate (nmol/L)**	82.0±5.8	82.6±10.0	35.5±6.0^*#^	107.9±5.8	102.4±15.2	19.6±3.5^*#^
**Plasma cobalamin (pmol/L)**	408.5±63.1	397.4±38.3	239.1±34.5*^#^	474.4±43.7	357.5±30.2	303.4±47.2*
**Cortex SAM (pmol/mg)**	118.2±12.5	96.5±7.5	87.7±6.5	100.5±6.7	97.2±11.2	95.0±12.3
**Cortex SAH (pmol/mg)**	55.3±7.7	43.1±5.7	55.3±8.1	52.9±7.7	37.5±3.6	52.2±12.9
**SAM/SAH ratio**	2.3±0.3	2.3±0.2	2.0±0.3	2.1±0.2	2.6±0.1	2.3±0.5
**Cortex total GSH (nmol/mg)**	5.6±0.5	7.6±0.7*^#^	4.6±0.6	8.9±0.7^§^	7.4±0.9	5.2±0.6*^#^
**Cortex reduced GSH (nmol/mg)**	4.6±0.5	6.6±0.6*^#^	3.4±0.5	7.3±0.8	6.3±0.9	3.6±0.4*^#^
**Cortex GSSG (nmol/mg)**	0.5±0.1	0.5±0.1	0.6±0.1	0.8±0.1	0.5±0.1	0.8±0.1
**GSH/GSSG ratio**	11.9±1.2	15.7±2.4	6.4±1.2*^#^	10.6±1.9	11.3±2.1	5.4±1.0*^#^

n = 6–8; PND  =  postnatal day; CT  =  control; DP  =  deficient diet during pregnancy; DPL  =  deficient during pregnancy and lactation; Hcy  =  homocysteine; Cys  =  Cysteine; GSH  =  glutathione; GSSG  =  oxidized glutathione; SAM  =  S-adenosylmethionine; SAH  =  S-adenosylhomocysteine. *ANOVA*. Data are presented as the mean ± standard error. ^*^Different from the CT group (*P*≤0.05). ^#^Difference between DP and DPL groups (*P*≤0.05). *t test*, § Difference between female and male control (*P*≤0.05).

To investigate whether a maternal Vitamin B-deficient diet during breast-feeding could affect methyl metabolism in the offspring's brain cortex, we quantified SAM and SAH concentrations at PND 28. The concentrations of brain cortex SAM, SAH and SAM/SAH ratio did not change in either female (*P* = 0.075, *P* = 0.0706 and *P* = 0.588, respectively) and male (*P* = 0.930, *P* = 0.469 and *P* = 0.565, respectively) offspring. However, decreases in total GSH, reduced GSH, and GSH/GSSG ratio were observed in the brain cortex of males in the DPL group when compared to the DP (*P* = 0.047, *P* = 0.013 and *P* = 0.028, respectively) and CT (*P* = 0.002, *P* = 0.009 and *P* = 0.049, respectively) groups. No significant difference in GSSG concentration was observed between groups (*P* = 0.200). In females, an increase of total GSH and reduced GSH was observed in the DP group when compared to the DPL (P = 0.003 and *P*<0.001) and CT (*P* = 0.033 and *P* = 0.014) groups, respectively. Alternatively, the GSH/GSSG ratio showed a significant decrease in the DPL group when compared to the DP (*P*<0.001) and CT (*P* = 0.027) groups. No change was observed in GSSG (*P* = 0.396) concentration in females. Considering genders, a statistically significant difference in total GSH concentration was observed between females and males in PND 28 offspring from the control group (*P*<0.001) (Table 2).

Exposure to a deficient diet during early development influenced the methyl pool, Hcy and GSH metabolism. Therefore, we investigated whether these changes persisted after weaning and exposition to a standard diet. Female offspring were analyzed at PND 90, and no changes were observed in plasma Hcy (*P* = 0.671), Cys (*P* = 0.409), total GSH (*P* = 0.451), folate (*P* = 0.648) or cobalamin (*P* = 0.102). As for the brain cortex, there were also no significant differences in SAM (P = 0.712), SAH (*P* = 0.773), SAM/SAH ratio (*P* = 0.620), total GSH (*P* = 0.430), reduced GSH (*P* = 0.996), GSSG (*P* = 0.061) or GSH/GSSG ratio (*P* = 0.290). In males, there were no significant differences in plasma Hcy (*P* = 0.084), Cys (*P* = 0.769), total GSH (*P* = 0.193), folate (*P* = 0.071) or cobalamin (*P* = 0.501). For the brain cortex, the levels of SAM (P = 0.684), SAH (P = 0.845), SAM/SAH ratio (P = 0.683), total GSH (P = 0.220), reduced GSH (P = 0.154), GSSG (P = 0.508) and GSH/GSSG ratio (P = 0.235) were not altered (Table 3).

**Table 3:** Effects of a Vitamin B-deficient diet during pregnancy and pregnancy/lactation on plasma and brain cortex parameters in offspring at PND 90.

PND 90	Female			Male		
	CT	DP	DPL	CT	DP	DPL
**Plasma Hcy (μmol/L)**	7.1±0.7	7.0±0.3	7.6±0.5	5.2±0.6	3.7±0.2	4.7±0.5
**Plasma Cys (μmol/L)**	379.3±15.1	386.3±10.4	409.1±21.2	387.8±19.8	416.8±14.8	408.5±40.8
**Plasma GSH (μmol/L)**	114.5±11.5	117.4±9.1	102.3±4.1	142.8±8.4	131.6±10.4	161.4±18.7
**Plasma folate (nmol/L)**	64.8±2.50	65.7±4.7	70.0±4.7	83.4±2.6	76.5±5.4	100.7±11.5
**Plasma cobalamin (pmol/L)**	169.8±27.1	310.5±47.9	290.1±61.7	276.6±41.2	298.6±31.1	350.0±59.8
**Cortex SAM (pmol/mg)**	48.6±2.4	50.5±2.9	47.2±3.1	45.0±3.1	42.5±2.8	40.5±5.0
**Cortex SAH (pmol/mg)**	33.2±1.2	34.5±1.4	34.2±1.6	31.1±1.8	31.1±2.0	29.4±3.2
**SAM/SAH ratio**	1.5±0.1	1.5±0.0	1.4±0.1	1.5±0.2	1.4±0.0	1.4±0.1
**Cortex total GSH (nmol/mg)**	4.1±0.2	4.0±0.2	4.5±0.3	4.6±0.3	4.3±0.3	3.8±0.4
**Cortex reduced GSH (nmol/mg)**	2.2±0.2	2.2±0.1	2.2±0.2	2.4±0.1	2.0±0.2	1.8±0.2
**Cortex GSSG (nmol/mg)**	1.0±0.0	0.9±0.0	1.1±0.1	1.1±0.1	1.1±0.1	1.0±0.1
**GSH/GSSG ratio**	2.3±0.2	2.4±0.1	2.0±0.2	2.2±0.2	1.8±0.1	1.8±0.2

n = 6–8; PND  =  postnatal day; CT  =  control; DP  =  deficient diet during pregnancy; DPL  =  deficient during pregnancy and lactation; Hcy  =  homocysteine; Cys  =  Cysteine; GSH  =  glutathione; GSSG  =  oxidized glutathione; SAM  =  S-adenosylmethionine; SAH  =  S-adenosylhomocysteine. *ANOVA.* Data are presented as the mean ± standard error.

Considering that aging is associated with the occurrence of metabolic and neurodegenerative diseases and that these conditions can be linked to maternal nutrition during pregnancy, we analyzed offspring at PND 210. The female offspring showed no differences in plasma Hcy (*P* = 0.146), Cys (*P* = 0.699), total GSH (*P* = 0.807), folate (*P* = 0.515) or in cobalamin (*P* = 0.240). The brain cortex concentration of SAM (*P* = 0.837), SAH (*P* = 0.711) and SAM/SAH ratio (*P* = 0.902) also remained unchanged. There were also no changes in brain cortex concentrations of total GSH (*P* = 0.980), reduced GSH (*P* = 0.682), GSSG (*P* = 0.380) or GSH/GSSG ratio (*P* = 0.374). In male offspring at PND 210, there were decreases in plasma folate and brain cortex SAM concentrations in the DPL group when compared to the DP (*P* = 0.014 and *P* = 0.012) and CT (*P* = 0.019 and *P* = 0.006) groups, respectively. Moreover, there was a decrease in plasma GSH concentration in both the DPL (*P* = 0.026) and DP (*P* = 0.026) groups when compared to the CT group. No differences were observed in the brain cortex concentration of SAH (*P* = 0.283), SAM/SAH ratio (*P* = 0.863), total GSH (*P* = 0.905), reduced GSH (*P* = 0.779), GSSG (*P* = 0.870) and GSH/GSSG ratio (*P* = 0.880), or the plasma concentrations of Hcy (*P* = 0.662), Cys (*P* = 0.873) and cobalamin (*P* = 0.841). The plasma Hcy of males at PND 210 were positively correlated (*P*<0.05) with brain cortex levels of SAM and SAH (*r* = 0.92 and *r* = 0.96, respectively). A positive correlation was also observed between plasma Hcy and brain cortex total GSH concentrations (*r* = 1.00, *P*<0.05). However, a negative correlation was observed between plasma GSH and brain cortex total GSH concentration (*r* = 0.48, *P*<0.05). Regarding the plasma Hcy concentration, a statistically significant difference was observed between genders in PND 210 offspring (*P*<0.001). Still considering metabolites related to Hcy metabolism, plasma GSH differences can also be observed in these animals (*P*<0.001, Table 4).

**Table 4:** Effects of a Vitamin B-deficient diet during pregnancy and pregnancy/lactation on plasma and brain cortex parameters in offspring at PND 210.

PND 210	Female			Male		
	CT	DP	DPL	CT	DP	DPL
**Plasma Hcy (μmol/L)**	8.1±0.8	6.7±0.8	9.0±0.8	3.0±0.4^§^	3.4±0.2	3.1±0.3
**Plasma Cys (μmol/L)**	382.1±19.4	379.2±13.3	396.4±11.7	395.2±15.8	387.8±27.9	393.1±17.4
**Plasma GSH (μmol/L)**	77.3±4.8	74.7±3.3	73.6±4.0	126.1±8.5^§^	99.5±8.4*	97.6±6.5*
**Plasma folate (nmol/L)**	58.4±3.7	67.9±15.8	75.6±8.8	88.1±6.1	87.6±2.0	67.4±7.4^*#^
**Plasma cobalamin (pmol/L)**	305.0±23.6	338.9±3.3	285.3±24.7	292.3± 65.0	256.7±32.0	297.0±56.7
**Cortex SAM (pmol/mg)**	46.4±3.5	42.6±7.0	47.0±1.6	52.7±1.0	51.9±1.6	46.1±1.6*^#^
**Cortex SAH (pmol/mg)**	38.8±1.9	40.6±1.3	40.9±2.3	40.2±1.0	39.1±2.2	35.8±2.1
**SAM/SAH ratio**	1.2±0.1	1.2±0.1	1.2±0.1	1.3±0.1	1.4±0.1	1.2±0.1
**Cortex total GSH (nmol/mg)**	5.4±0.2	5.4±0.3	5.4±0.2	6.2±0.2	6.1±0.3	6.3±0.2
**Cortex reduced GSH (nmol/mg)**	4.1±0.2	3.9±0.3	4.2±0.2	4.7±0.1	4.7±0.2	4.9±0.3
**Cortex GSSG (nmol/mg)**	0.7±0.1	0.8±0.2	0.6±0.1	0.7±0.1	0.7±0.1	0.7±0.1
**GSH/GSSG ratio**	7.3±1.3	5.8±0.9	8.2±1.2	7.0±1.0	7.2±0.8	7.8±1.3

n = 6–8; PND  =  postnatal day; CT  =  control; DP  =  deficient diet during pregnancy; DPL  =  deficient during pregnancy and lactation; Hcy  =  homocysteine; Cys  =  Cysteine; GSH  =  glutathione; GSSG  =  oxidized glutathione; SAM  =  S-adenosylmethionine; SAH  =  S-adenosylhomocysteine. *ANOVA*. Data are presented as the mean ± standard error. ^*^Different from the CT group (*P*≤0.05). ^#^Difference between DP and DPL groups (*P*≤0.05). *t test*, § Difference between female and male control (*P*≤0.05).

## Discussion

The placenta is the interface between fetal and maternal circulation and plays a critical role in the regulation of fetal growth and development through controlled nutrient supply. In this context, there is an increase of cellular proliferation and one-carbon metabolism resulting from placental development, fetal growth, uterine enlargement and expansion of blood volume [27]. As referred to above, SAM/SAH ratio is used as an index of cellular methylation and SAH is an inhibitor of most SAM-dependent methyltransferases. These include the DNA methyltransferases. In fact, increased Hcy and SAH levels, decreased SAM/SAH ratios and global DNA hypomethylation were reported in healthy humans [28]. Data published by Kim *et al*. [21] indicated that a folate-deficient diet supplemented by Hcy decreased SAM concentration and SAM/SAH ratio in both the placenta and the liver of pregnant rats, and these changes were linked to the decrease in DNA methylation. In humans, a recent study reported that increased Hcy concentration changes placental global methylation levels in women with pregnancy complications, such as preeclampsia and preterm delivery [29]. The observed abnormal global DNA methylation might reflect changes in normal temporal regulation of gene expression that is crucial for the optimal development of the fetus and may have implications for many metabolic processes. In our study, we observed that a similar diet manipulation also alters methyl metabolism in newborn brains. Decreases in SAM concentration and SAM/SAH ratio in the total brain of male offspring were observed. In females, we also observed a decrease in SAM/SAH ratio; however, this change was due to an increase in SAH concentration instead of a reduction of SAM concentration, as seen in the males. These results show that, although male and female newborns exhibited a decrease in SAM/SAH ratio, there is a crucial difference in the maternal-fetal interaction between genders. A study published by Guerra-Shinohara *et al*. [30] showed that, in humans, low maternal concentrations of cobalamin and folate and elevated plasma Hcy concentrations were associated with a decrease in the plasma SAM/SAH ratio in newborns; however, the gender specificity of this difference was not analyzed. Our findings showing gender differences in mice indicate the need for further studies to conclude whether these differences in the concentrations of SAM and SAH, among young males and females (CT and DP groups), are due to a differential redirection of methionine or vitamins by mothers or to differential response of the fetus (male or female) to vitamin deficiency.

A pioneering study published by Barker and Osmond (1986) showed a relation between nutrition and growth before birth and during early childhood and the development of chronic diseases later in life [16]. Another notable work by Gueant (2011) revealed that a methyl donor deficiency produces detrimental effects on fatty acid oxidation and energy metabolism of myocardium. These alterations should be clinically evaluated as a potential causal and/or aggravating metabolic condition of perinatal cardiomyopathies [31]. Additionally, maternal malnutrition is suggested to be a major non–genetic factor that can lead to a disturbed brain development, and it has been proposed that increased oxidative stress during pregnancy may be a result of under or over nutrition [32]. GSH has a vital function in protecting tissues against the degenerating effects of oxidative damage by scavenging free radicals from endogenous or exogenous agents [33], [34], and the GSH/GSSG ratio is considered to be a sensitive indicator of the cellular redox state [35]. With this fact in mind, in our study, the high Hcy concentration in maternal-fetal circulation could lead to oxidative stress and, as a consequence, a decreased efficacy of defense mechanisms such as GSH availability. Additionally, total GSH concentration could increase as a result of maternal programming by epigenetic response to high plasma Hcy and poor remethylation. In newborns (DP) from deficient diet dams, we observed no change in brain concentrations of total GSH and fractions (GSH and GSSG). Alternatively, increased plasma Hcy concentration seems to be associated with a decrease in total GSH and reduced GSH and thus a deficit in antioxidant capacity (GSH/GSSG ratio) in the brain cortex of DPL males at PND 28. In females, we observed the same decrease in antioxidant capacity in the DPL group when compared to the CT and DP groups. However, when total GSH and reduced GSH were analyzed, we observed an increase in the DP group compared to the CT and DPL groups. No significant differences in the concentrations of SAM, SAH and SAM/SAH ratio were observed in females and males mice at PND 28. However, Blaise *et al*. [22] has shown an increase in SAH concentration and decrease in SAM/SAH ratio in the brain of rat offspring submitted to a deficient diet during pregnancy and lactation. A study published by Jathar *et al*. [36] showed no change in cobalamin concentration in the breast milk of lacto-vegetarian mothers compared to non-vegetarians, even though a decrease in the serum cobalamin of lacto-vegetarians mothers have been reported. In our study, we did not analyze the breast milk composition, but we observed an increase of approximately 120% in the plasma Hcy concentration of the offspring (PND 5) breastfed by dams fed the deficient diet. Moreover, there was a deficit in antioxidant capacities in the brain cortex of DPL offspring at PND 28. Additionally, a decrease in plasma folate and cobalamin was observed in the DPL offspring (PND 28) from dams fed the deficient diet during gestation and lactation.

In our study, we observed an increase in plasma folate concentration in DPL males (PND 90), but the difference did not reach statistical significance (*P* = 0.071). A study performed in rats and published by Blaise *et al*. [22] showed an increase in plasma folate concentration in these animals at PND 80. Considering fetal development, the exposure to a diet deficient in methyl donor groups during pregnancy or pregnancy/lactation may have caused a metabolic variation throughout life in response to the initial vitamin deficiency. Furthermore, the data showed that there is a difference in Hcy metabolism between females and males, which is implied by the observed decrease in plasma Hcy concentrations throughout life in only males. An article published by Vitvitsky *et al*. [37] suggests that testosterone-mediated up-regulation of renal cystathionine-β-synthase levels in mice contributes to lower tHcy levels in males. At older an age (PND 210), a decrease in plasma total GSH concentration was observed only in females. This data corroborates with another study developed by Hirayama *et al*. [38] and can be directly related to decreased transsulfuration in females. Moreover, our data strengthen the evidence that there is a difference in the maternal-fetal interaction between males and females, as implied by the different patterns of metabolic responses found during development. At PND 0, increased SAH and decreased SAM concentrations in the brain of DP offspring were observed in females and males, respectively. DPL males at PND 210 showed a decrease in both plasma folate and brain cortex SAM concentrations. Additionally, there was also a decrease in plasma total GSH in both DP and DPL males. The plasma Hcy of males at PND 210 were positively correlated with brain cortex SAM, SAH and total GSH concentrations. However, a negative correlation was observed between plasma GSH and brain cortex total GSH concentration.

Other studies have also shown gender differences in response to maternal manipulation or environmental exposure (i.e., pollution) [39], [40]. Recent studies on epigenetic mechanisms show that males may be more likely to exhibit changes in adulthood than females due to epigenetic marks that have occurred in the embryonic period [41]. Furthermore, based on evolutionary theories, we can say that the differences between genders in response to an “adverse environment” are the result of selective pressures with potential benefits to females observed among mammals [42]. The predominant agent in this case is the reproductive role of the female that involves the offspring's care, an activity that requires a high consumption of energy, especially considering pregnancy and lactation [43], [44]. However, in species where the male has a greater role in the care of offspring or if the insult is severe, these differences are minimized [42], [45].

In conclusion, a Vitamin B-deficient diet during pregnancy alters SAM and SAH metabolism in the brain of newborn mice. Moreover, this diet increases plasma Hcy and decreases the antioxidant capacities of the brain cortex in offspring breastfed by dams fed the deficient diet during lactation. Nutritional cobalamin deficiency due to maternal deficiency might be a serious health problem in infants; therefore, screening and supplementation of pregnant and lactating women to prevent infantile cobalamin deficiency should be considered [46]. The early exposition to methyl group deficiency seems to impact female and male offspring differently. Moreover, these effects persist in the offspring in a long lasting manner; i.e., males at PND 210 showed a decrease in plasma folate, brain cortex SAM (DPL) and in plasma total GSH concentrations (DP and DPL). Therefore, the decreased plasma GSH concentration may reflect redox changes in tissues, and the imbalance in the availability of methyl groups may alter the programming of genes involved in the etiology of age-related degenerative diseases.

## Materials and Methods

### Animal Treatment Protocols

Animal experiments were performed on Swiss mice and conducted according to the Guide for the Care and Use of Laboratory Animals (8th edition, National Academy Press, Washington D. C., 2011) and were approved by the Institutional Animal Care and Use Committee of the Universidade Federal de São Paulo (#1169/08). Adult female mice were maintained under standard laboratory conditions, on a 12-hour light/dark cycle, with food and water available *ad libitum*. One month before pregnancy, twenty-eight female mice were distributed into the following groups (n = 14 per group): a) standard diet (AIN-93M) and, b) a diet deficient of vitamins B12 (2.37 μg/kg), B2 (0.938 mg/kg), folate (0.290 mg/kg), and choline (0.1736 mg/kg), (LabDiet, St. Louis, MO). Male mice were placed in the females' home cages for mating and gestational day zero was determined by confirming the presence of sperm in the content of vaginal smear. This vitamin B and choline deficient diet creates a significant methyl group deficit in the fetus.

After birth, offspring were distributed into three groups: control (CT), deficient diet during pregnancy and lactation (DPL) and deficient diet during pregnancy (DP). The dams of offspring CT and DPL received, respectively, standard and deficient diet during pregnancy and lactation. The dams of offspring DP received deficient diet during pregnancy, but controls dams adopted this offspring during lactation (receiving standard diet). To normalize the effect of adopting suffered by the DP group, maternities were exchanged within each group, CT and DPL, and adjusted the litter for n = 8 animals per dams (n = 4, females; n = 4, males) in all groups. All offspring groups were breastfed until PND 28 and after weaning, all of them received a standard diet.

### Sample Collection

Males and females were euthanized by decapitation at different developmental stages (PND 0, 5, 28, 90 or 210). The analysis on these time points allows us to infer the consequences of vitamin B and choline deficiency during pregnancy and pregnancy/lactation in the short term (PND 0, 5 and 28), and possible changes that arise or remain even after the introduction of the standard diet (post-weaning) can be observed in the medium and long term, PND 90 and 210, respectively. Considering that gender differences were widely described in the literature for several parameters and also after manipulation during pregnancy and postnatal period, males and females were analyzed separately in this study.

Blood was collected in tubes (Becton Dickinson, New England, UK) containing ethylenediaminetetraacetic acid (EDTA) and stored on ice, and up to 90 minutes, centrifuged at 3000 rpm for 10 minutes at 4 °C. Plasma aliquots of all stages, except PND 0, were stored at −80°C for Hcy, Cys, GSH, folate and cobalamin measurements. The whole brain was collected, rapidly harvested and stored at −80°C for subsequent SAM, SAH, total GSH and reduced GSH measurements/quantification.

### Plasma measurements

Plasma Hcy, Cys and total GSH were analyzed by high-performance liquid chromatography (HPLC) through fluorescence detection and isocratic elution. The method was developed by Pfeiffer *et al*. [47] with slight modifications: column C18 Luna (5 μm, 150 mm×4.6 mm), mobile phase (0.06 M sodium acetate, 0.5% acetic acid, pH 4.7 (adjusted with acetic acid), 2% methanol) and flow rate of 1.1 mL/min. The retention time was 3.6 minutes for Cys; 5.2 for Hcy and 9.0 for GSH [48].

Plasma cobalamin concentration was determined by ELISA using the CUSABIO kit, and the plasma folate concentration was quantified by microbiological assay [49].

### Tissue measurements

Total brain or total dissected cortex was homogenized in PBS using a tissue homogenator (T10 basic IKA, Staufen, Germany).

For SAM and SAH measurements, the protein and debris were precipitated from total homogenate tissue with HClO_4_ and centrifuged. The supernatant was injected into the column C18 LiChroCart (5 μm, 250 mm×4 mm). The mobile phase was applied at a flow rate of 1 mL/min and consisted of 50 mM sodium phosphate (pH 2.8), 10 mM heptan sulfonate, and 10% acetonitrile. The UV detector had a wavelength of 254 nm. The retention time was 8.7 minutes for SAH and 13.6 minutes for SAM, a technique adapted from Blaise *et al*.[50].

Total brain GSH measurements were performed using the same method previously described for measuring total plasma GSH. For reduced GSH quantification, the reducing agent was not added, and the concentrations were calculated.

### Statistical Analyses

Analyses of dams, offspring at PND 0 and PND 5, and females and males controls were performed using *t test* for independent groups. For the analysis of offspring at PND 28, PND 90 and PND 210 we performed analysis of variance (ANOVA) followed by *post hoc* Fisher's test when necessary. For finding out significant relation between two variables, Pearson's correlation was used. Data were presented as the mean ± standard error. The level of significance was *P*≤0.05. The program STATISTICA 8.0 was used to perform the analysis.
